# The Complete Mitogenome of *Toxocara vitulorum*: Novel In-Sights into the Phylogenetics in Toxocaridae

**DOI:** 10.3390/ani12243546

**Published:** 2022-12-15

**Authors:** Yue Xie, Lidan Wang, Yijun Chen, Zhao Wang, Pengchen Zhu, Zun Hu, Xinfeng Han, Zhisheng Wang, Xuan Zhou, Zhicai Zuo

**Affiliations:** 1 Department of Parasitology, College of Veterinary Medicine, Sichuan Agricultural University, Wenjiang 611130, China; 2 Department of Food Technology and Science, College of Food Science, Shanghai Ocean University, Shanghai 201306, China; 3 Key Laboratory of Animal Disease and Human Health of Sichuan Province, College of Veterinary Medicine, Sichuan Agricultural University, Chengdu 611130, China; 4 Animal Nutrition Institute, Sichuan Agricultural University, Chengdu 611130, China

**Keywords:** roundworms, *Toxocara*, mitochondrial genome, phylogenetic relationships

## Abstract

**Simple Summary:**

*Toxocara vitulorum* is a common parasitic nematode of cattle and buffalos, and represents a severe health threat and economic burden on both populations. Poor understanding the epidemiology, population genetics, and molecular ecology of this nematode negative affects the prevention and control of *T. vitulorum*, and the mitogenome can yield a foundation for studying these areas. Herein, the first whole mitogenome of *T. vitulorum* was sequenced and characterized with bioinformatic pipeline analyses. Comparative mitogenomics and phylogenies defined a novel sister-species relationship of *T. vitulorum* with the congeneric species in the Toxocaridae, and supported nad4 and nad6 genes as new genetic markers for phylogenetic and evolutionary studies of the Ascaridida species. These results should contribute to a better understanding of phylogenetic relationships of Toxocaridae species, and also provide the resource of markers for population genetics, systematics, and epidemiology of this bovine nematode.

**Abstract:**

*Toxocara vitulorum* (Ascaridida: Nematoda) is one of the most common intestinal nematodes of cattle and buffalos and, therefore, represents a serious threat to their populations worldwide. Despite its significance in veterinary health the epidemiology, population genetics, and molecular ecology of this nematode remain poorly understood. The mitogenome can yield a foundation for studying these areas and assist in the surveillance and control of *T. vitulorum*. Herein, the first whole mitogenome of *T. vitulorum* was sequenced utilizing Illumina technology and characterized with bioinformatic pipeline analyses. The entire genome of *T. vitulorum* was 15,045 bp in length and contained 12 protein-coding genes (PCGs), 22 transfer RNAs (tRNAs), and two ribosomal RNAs (rRNAs). The gene arrangement (GA) of *T. vitulorum* was similar to those of other *Toxocara* species under GA3. The whole genome showed significant levels of AT and GC skew. Comparative mitogenomics including sequence identities, Ka/Ks, and sliding window analysis, indicated a purifying selection of 12 PCGs with cox1 and nad6 having the lowest and highest evolutionary rate, respectively. Whole amino acid sequence-based phylogenetic analysis supported a novel sister-species relationship of *T. vitulorum* with the congeneric species *Toxocara canis*, *Toxocara cati*, and *Toxocara malaysiensis* in the family Toxocaridae. Further, 12 (PCGs) single gene-based phylogenies suggested that nad4 and nad6 genes shared same topological trees with that of the whole genome, suggesting that these genes were suitable as novel genetic markers for phylogenetic and evolutionary studies of Ascaridida species. This complete mitogenome of *T. vitulorum* refined phylogenetic relationships in Toxocaridae and provided the resource of markers for population genetics, systematics, and epidemiology of this bovine nematode.

## 1. Introduction

*Toxocara vitulorum* is one of the most common intestinal nematodes of cattle and buffalos, and poses a serious threat to younger populations by influencing feed efficiency and performance in the humid tropics [1]. *T. vitulorum* adult parasites live in the small intestine of the calves, and mate and reproduce unembryonated eggs in the surroundings [2,3]. Adult bovines become infected by ingestion of the infective second-stage (L2)-contained eggs. Then, the L2 larvae hatch and migrate to muscles, liver, kidneys, and other viscera for development of hypobiotic larvae [4]. These arrested larvae are activated and re-migrate to the mammary gland and the utero around the time of parturition, so calves can be infected with *T. vitulorum* through amniotic fluid or colostrum and milk from infected mothers [5]. Alternatively, calves can also be infected by ingestion of infective eggs from the surrounding [6,7]. The larvae mature in the small intestine of calves and shed eggs in the feces. Clinical symptoms in calves include anemia, diarrhea, weight loss, and obstruction of the small intestine, even death [8]. Moreover, farmers who drink raw cattle or buffaloes milk are likely to infect *T. vitulorum* and suffer visceral larval migraines (VLM) [9]. Although *T. vitulorum* is widespread throughout the world, it is frequently found in tropical and subtropical areas, especially those with high temperatures [10]. For example, the prevalence of *T. vitulorum* was 17.6% in northeast Florida [8], 22.6% in LAO PDR [11], 28.4% in Egypt [9], and 37.5% in Pakistan [12]; in contrast, sporadic infection reports of *T. vitulorum* were documented in Germany [13], Netherlands [14], the UK [15], and Belgium [16].

Customarily, *T. vitulorum* has been identified and classified based on its morphological features and particular host preference [17]. However, the taxonomic investigation of *T. vitulorum* was questioned due to significant limitations in traditional methods for the accurate identification and differentiation from other *Toxocara* species, particularly at the larval or egg stages [18]. Since molecular characterization techniques can be used to establish the genetic makeup and phylogenetic relationships with high sensitivity and specificity [19], it is important and necessary to create precise identification techniques for identification and differentiation of *T. vitulorum*. In fact, some molecular markers, for instance, the genes ribosomal protein 18 (18S), large subunit 28S region (28S), first internal transcribed spacer (ITS-1), and second internal transcribed spacer region (ITS-2), have been used to distinguish *T. vitulorum* from other related species [20,21,22,23]. Additionally, the mitochondrial (mt) DNA has also attracted more and more attention and proven more suitable than the ribosomal sequences for species identification due to its matrilineal inheritance, lack of recombination, and rapid rate of evolution [24,25,26,27]. For example, mt genes atp6, nad1, and cox1 were also used to identify *T. vitulorum* [9,23,28,29]. Nevertheless, compared with single gene or partial genetic genes, analysis based on the full mitogenome provides particularly effective information in the distinctiveness of species and interspecies sequence variability and therefore could yield novel opinions into evolutionary and phylogenetic-based analyses of nematode parasites. However, no complete mitogenome of *T. vitulorum* has been reported to date [30], in the present study, the complete mitogenome of *T. vitulorum* was sequenced and assembled by Illumina technology for the first time, which enabled the entire mitochondrial genome-based phylogenetic research and refined the phylogenetic relationships of *T. vitulorum* in the whole *Toxocara* and of the genus *Toxocara* in the order Ascaridida.

## 2. Materials and Methods

### 2.1. Parasite

In September 2021, several yak calves died of diarrhea, anorexia, and dehydration in Hongyuan, Sichuan Province of China. After postmortem examinations, a large number of adult nematodes were found in the small intestines. These nematode specimens (*n* = 50) were subsequently brought to Sichuan Agricultural University (Sichuan, China) for species identification and characterization. After washing in physiological saline, all nematodes were preliminarily identified as *T. vitulorum* based on morphologies (Figure S1), as described elsewhere [5]. Then, these worms were frozen in 50% (*v*/*v*) ethanol and kept at −20 °C for molecular study.

### 2.2. DNA Isolation

Total genomic DNA was extracted from a small portion (>1.5 cm) of one specimen using the Genomic DNA Extraction Kit Ver. 3.0 (TaKaRa, Shigo, Japan). PCR amplification targeting the ITS2 sequence of rDNA was used to confirm the identity of this worm by comparison with that previously reported for *T. vitulorum* (GenBank accession number EU189085.1). Once again, this nematode was recognized as *T. vitulorum* with >99.5% sequence identity.

### 2.3. Genome Assembly and Annotation

By BerryGenomics Company (Beijing, China), an Illumina TruSeq library was created using genomic DNA, with an average insert size of 480 bp, and sequenced on a full runoff (500 cycles) and paired-end (250 bp reads) on the Illumina Hiseq2500 platform. In total, 3.5 Gb of clean data were obtained after filtering short and low-quality reads with poly-Ns (>15 bp Ns) or >75 bp bases with quality score ≤ 3. These clean reads were assembled using IDBA-UD [31] with the parameters (similarity threshold 98% and minimum and maximum K values of 80 and 240 bp, respectively). To check the accuracy of the assembly, clean reads were mapped into the acquired mitogenome sequences using Geneious 10.1.3 [32]. At the same time, this assembled mitogenome was also validated by PCR amplifications using eight overlapping segments (ranging in length from 1.55 kb to 2.88 kb), that were designed on the basis of the alignments of the relatively conserved regions of the available *Toxocara* mitogenomes. The corresponding primer sequences are shown in Table S1. PCR reactions were performed in a 25 μL reaction volume containing 2 μL genomic DNA, 2×*TransTaq^®^* HiFi PCR SuperMix (TaKaRa, Japan), 0.1 μM of each primer and ddH_2_O. PCR conditions were 4 min denaturation at 94 °C, followed by 35 cycles of 40 s at 94 °C, 45 s at 46~54 °C and 2~4 min at 68 °C according to the Tm values and the product lengths, with a final extension at 68 °C for 10 min. After agarose gel detections, all target amplicons were sequenced either directly or following sub-cloning into the pMD19-T vector (TaKaRa, Japan). Each amplicon was sequenced three times to ensure maximum accuracy. The final version of *T. vitulorum* mitogenome was also annotated by manual alignments with published full mitogenome sequences of the *Toxocara canis*, *Toxocara cati*, and *Toxocara malaysiensis* [33] using the Clustal_X v.2.1 and online BLAST searcher through the NCBI website [34]. Transfer RNAs (tRNAs) and their secondary structures were predicted by using the online tRNAscan-SE Search Server [35]. MacVector v. 9.5 [36] was used to create the circular map. The complete mitogenome of *T. vitulorum* was deposited in GenBank under the accession number: OP466744.

### 2.4. Sequence Analysis

Primer v5.0 software [37] was used to infer and analyze the amino acid sequences of protein-coding genes (PCGs) based on the Invertebrate Mitochondrial Code, and MEGA 6.0 [38] was used to examine the codon use profiles. Further, the nucleotide skewness of *T. vitulorum* and their comparisons with 30 other nematodes in order Ascaridida (Table 1) were performed using the formulas [39]: AT skew = (A − T)/(A + T) and GC skew = (G − C)/(G + C). The nucleotide as well as amino acid sequences of each PCG and the concatenated PCGs obtained from aforementioned species (including *T. vitulorum*) were aligned using the MEGA 6.0 software. Based on pairwise alignments, the DNASTAR v15.3 program [40] was used to compute the identity percentages of nucleotide and amino acid sequences, and the synonymous (Ks) and non-synonymous (Ka) substitution rates among them were determined using DnaSP 6.12.03 [41]. Additionally, a sliding window analysis of 200 bp and a step size of 20 bp was also performed in DnaSP 6.12.03 to calculate the nucleotide divergence Pi among the mitogenomes of the order Ascaridida.

### 2.5. Phylogenetic Analysis

Phylogenies were inferred from either the concatenated amino acid sequence dataset of 12 PCGs or single amino acid dataset of each PCG of the *T. vitulorum* mitogenome. During the procedures, amino acid sequences were aligned with those of 26 other related nematodes (Table 1) using ClustalX and T-Coffee 7.81 [58], and the ambiguous regions (59 aa for the concatenated 12 PCGs and 0–32 aa for the single PCG) in the alignments removed using GBLOCKS 20.03 b [59]. Phylogenetic analyses were performed with the Maximum Parsimony (MP), Maximum likelihood (ML), and Bayesian inference (BI), using the Seuratoidea species *C. robustus* as the outgroup in each analysis. The MP analysis were constructed using PAUP* [60], and either the concatenated 12 PCGs dataset or multi-alignments of the amino acid sequence for each PCG were analyzed using equally weighted parsimony and heuristic searches with a tree-bisection-reconnection (TBR) branch-swapping. 1000 replicates of Wagner trees were chosen and five trees per replication were sampled, followed by the harvest of the optimal tree using Kishino-Hasegawa. Bootstrap resampling with 1000 replications was calculated for each nodal support. For ML analyses, the topological trees were constructed using PHYML 3.0 [61] under the LG +C4 + F + I (concatenated dataset of 12 PCGs) and LG + F + I (single PCG dataset) models of amino acid substitution selected with ProtTestn [62]. ML analyses were carried out using an ML + rapid bootstrap algorithm with 1000 replicates for concatenated datasets or 100,000 replicates for single gene dataset. In order to harvest a stable result, partitioned and non-partitioned ML analyses were repeated 20 times with different seeds. For BI analyses, Bayesian information criterion was used to select the optimal evolutionary models for mitogenomic data using ModelFinder [63]. The BI computations were achieved using MrBayes 3.2.7 [64], with four independent Markov chain, running for 2,000,000 (concatenated dataset of 12 PCGs) and 1,000,000 (single PCG dataset) metropolises coupled Monte Carlo generations, sampling a tree every 200 and 1000 generations. When the average standard deviation (SD) of the split frequencies reduced to below 0.01, the first 25% trees were discarded as ‘‘burn-in’’ and the remaining were used to compute Bayesian posterior probabilities (PPs). The evolutionary distance was estimated using the MrBayes order (aamodelpr = mixed) with default parameters. A consensus tree was obtained and visualized using Treeview X [65].

## 3. Results and Discussion

### 3.1. General Feature of T. vitulorum Mitogenome

**Figure 1:**
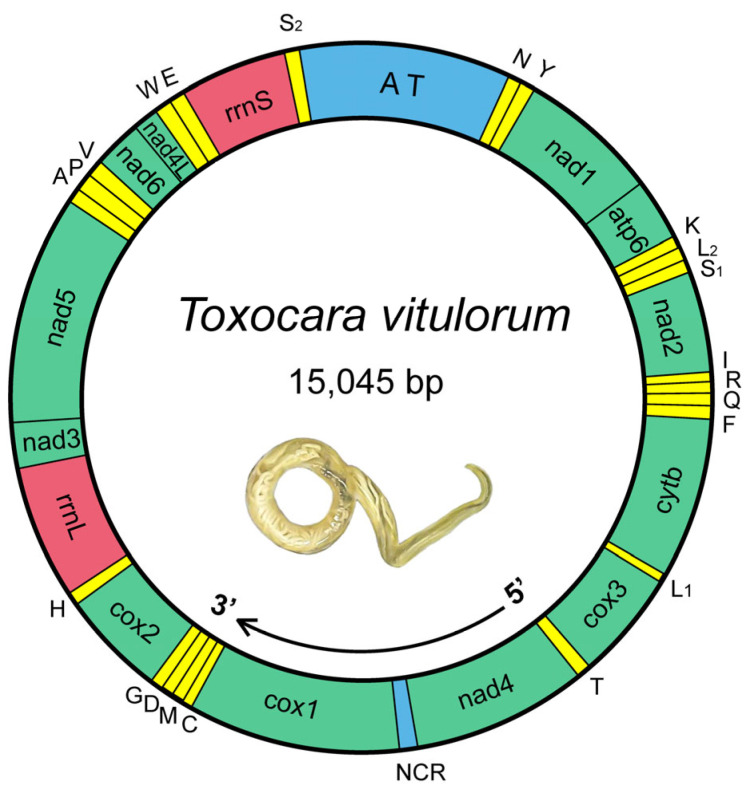
Circular map for *T. vitulorum* mitogenome. 36 genes follow standard nomenclature, including 22 tRNAs, which are denoted by one letter in accordance with the IPUC-IUB single-letter amino acid codes. The two leucine genes were differed by L1 and L2, and the two serine genes by S1 and S2. All the genes are present on the same strand and transcribed in the same direction (5′–3′), as indicated by the arrow. NCR indicates the non-coding region. AT indicates the AT-rich region.

The whole nucleotide sequence of mitogenome of *T. vitulorum* was determined to be 15,045 bp in size (Figure 1). It contained 36 genes, consisting of 12 PCGs (one subunit of the adenosine triphosphatase synthase, atp6; three subunits of cytochrome c oxidase, cox1-3; one subunit of cytochrome c-ubiquinol oxidoreductase, cytb; and seven subunits of nicotinamide dehydrogenase, nad1-6 and nad4L), 22 tRNAs (including two coding for leucine and two coding for serine), and 2 ribosomal RNAs (rRNAs) (small [rrnS] and large [rrnL] subunits), but lack an atp8 gene (Table 2), like other chromadorean nematode mitogenomes reported thus far [66]. All the genes were on the same strand and transcribed in the same direction, same as all previously characterized chromadorean nematodes [48]. The gene orders of the mitogenome followed the gene arrangement (GA3) and were similar to those of the other *Toxocara* members [56,67]. The mitogenome of the *T. vitulorum* contained seven overlapping regions, only one of them was 10 bp in length and located across the neighboring cox2 and trnH (His) genes, others were all 1 bp in length located between trnT (Thr) and nad1 genes, nad2 and trnI (Ile) genes, trnQ (Gln) and trnF (Phe) genes, trnD (Asp) and trnG (Gly) genes, nad6 and nad4L genes, trnW (Trp) and trnE (Glu) genes, respectively. Eleven intergenic spacers ranged in length from 1 to 1701 bp including one AT-rich region (also known as the control region) and one non-coding region (NCR), which were located between the trnS2 and trnN (Asn), and between cox1 and nad4, respectively, similar with other *Toxocara* species [33].

Among the mitogenomes of species in *Toxocara*, the PCGs accounted for the largest proportion, comprising 68.42~73.99% of the total size, followed by the RNA regions, accounting for 19.33~20.67% of the mitogenomes. Intergenic regions were the smallest parts of the mitogenomes, accounting for only 0.83~1.05% of the whole mitogenomes, showing that the mitogenomes of those organisms had rather compact structures (Figure S2). *T. vitulorum* was about 0.7~1.0 kb longer than the mitogenomes of other *Toxocara* species, in which the AT-rich region contributed the vast majority of expansion of the mitogenome. However, compared to other *Toxocara* species, the proportion of PCG regions was reduced in *T. vitulorum*. It may relate to an increased length in AT-rich region of *T. vitulorum*.

### 3.2. Nucleotide Composition and Codon Usage

**Figure 2:**
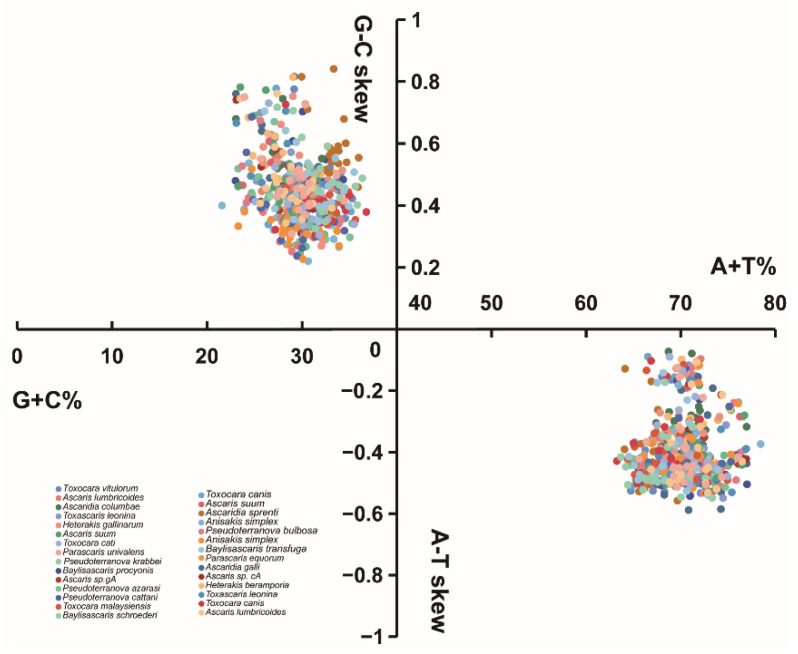
AT-skew vs. GC-skew of the different genetic positions of 29 mitogenomes. The X axis provides the A + T/G + C values, while the Y axis provides the skew values. For each species, the entire mitogenome, concatenated PCGs, concatenated tRNAs, concatenated rRNAs, the 1st, 2nd, 3rd, and 12 PCGs were separate calculated.

The overall nucleotide composition of the *T. vitulorum* mitogenome was 47.93% T, 8.34% C, 22.02% A, and 21.72% G, which came to the conclusion that T was the predominate nucleotide and C was the least favored. For the mitogenome of *T. vitulorum*, the concatenated PCGs had an A + T content of 66.93%, tRNAs had a value of 68.98%, rRNAs had a value of 72.22%, and the mitogenome as a whole had a value of 69.94%. Of the whole mitogenome, *T. vitulorum* was moderately G-skewed (GC-skew = 0.445) and T-skewed (AT-skew = −0.370), these values were similar to the skewness of *T. canis*, *T. cati*, and *T. malaysiensis* (Table S2), and among them, the mitogenome of *T. vitulorum* had the strongest bias towards T and G. Four parameters (AT-skew, GC-skew, A + T content, and G + C content) were used to make Figure 2 to compare nucleotide compositions of *T. vitulorum* with other nematodes. All nematode mitogenomes calculated in the figure, their AT-skews were negative, while GC-skews were positive, and they were rich in A and T, while the A + T contents of whole genomes ranged from 63.24% (*T. canis*) to 78.45% (*A. simplex*), and the G + C content were between 21.56% (*A. simplex*) and 36.76% (*T. canis*). Evidence from the investigations on the spontaneous deamination process of C and A during mammal mitogenome replication seemed to make a prediction that the strand compositional asymmetry could be attributed to the asymmetrical directional mutation pressure resulting from mitogenome replications of those nematodes including *T. vitulorum* [68,69].

**Figure 3:**
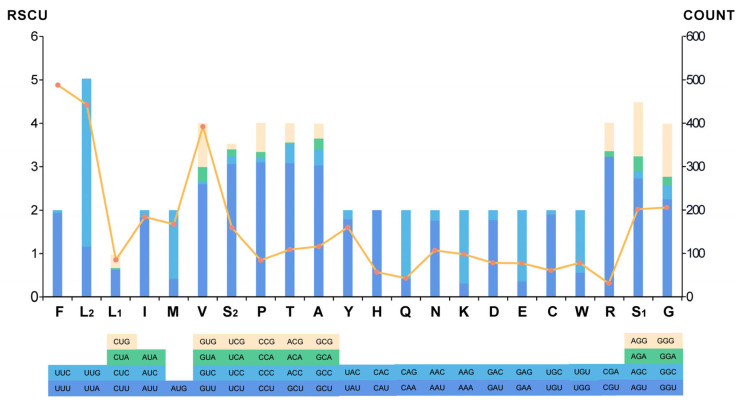
The RSCU and codon numbers in *T. vitulorum* mitogenome. Different color bars represent the RSCU of each codon; the codon numbers are indicated by the orange line graph (right Y axis scale).

For the codon usage, the preferred nucleotide usage at the third codon position of the *Toxocara* species PCGs reflected the total nucleotide content of the mitogenomes. At that position, the most frequently used nucleotide was T, whereas the least frequently use was C. We also calculated relative synonymous codon usage (RSCU) and codon counts in the mitogenome of *T. vitulorum* (Figure 3). It was evident that the most frequently used codon was UUG (RSCU = 3.87), followed by CGU (RSCU = 3.23) and CCU (RSCU = 3.10). The most frequently used amino acid was Phe (Count = 488), Leu (UUR) (Count = 442) and Val (Count = 392). The most frequently used start codon for the *T. vitulorum* is TTG (six genes; cox1, cox3, cytb, nad1, nad3, and nad6), followed by ATT (three genes; atp6, nad2, and nad4L), and GTG (nad4) and GTT (cox2) and ATG (nad5). Eight of the PCGs were predicted to use TAG (cox1, cox2, cox3, and nad4) or TAA (cytb, nad1, nad3, and nad5) as the termination codons, while the remaining genes (nad2, nad4L, nad5, and atp6) were deduced to end with an incomplete codon, such as T or TA.

### 3.3. tRNAs and rRNAs

**Figure 4:**
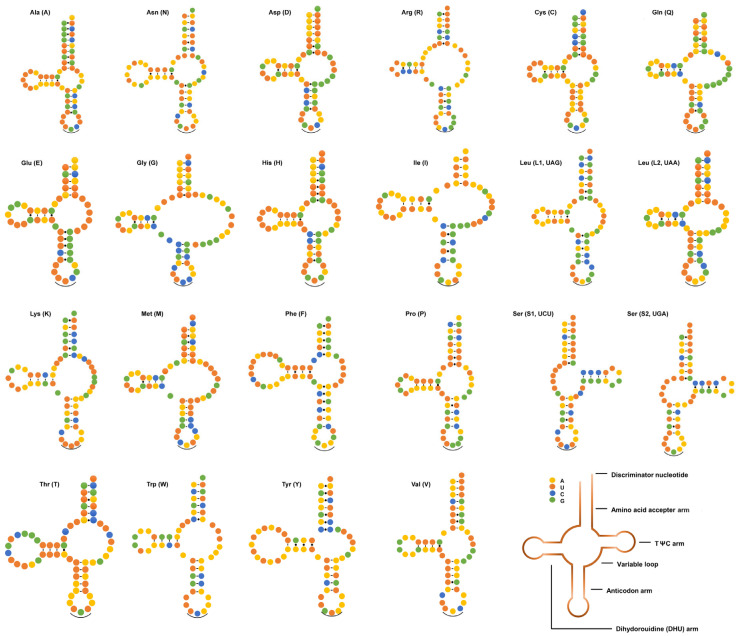
Predicted secondary structures of 22 tRNAs in *T. vitulorum* mitogenome. The tRNAs are labeled with their corresponding IPUC-IUB single-letter amino acid codes. Dashes (−) denote Watson–Crick bonds, dots (·) indicate mispaired nucleotide bonds, and (‿) indicate tRNA anticodons.

The mitogenome of *T. vitulorum* contained 22 tRNA genes, with lengths ranging from 52 bp (trnS [UGA]) to 72 bp (trnT) (see Table 2). The 22 tRNA secondary structures estimated (Figure 4) differed significantly from the typical cloverleaf-like structures reported in other metazoan mitogenomes but were identical to those of all other chromadorean nematodes investigated so far [57,70,71,72,73,74,75], with the exception of *T. spiralis* [75]. In addition, 22 anticodons (Table 1) of the *T. vitulorum* were as same as other species in the same genus [45,57]. For the mitogenome of *T. vitulorum*, the rrnS gene was 688 bp in length and the rrnL gene was 954 bp in length (Table 2). The rrnL was found between trnH and nad3, and rrnS was located between trnE and trnS2. The four PCGs in the mitogenome (nad3, nad5, nad6, and nad4L) kept two genes apart from one another (Figure 1). Their lengths were comparable to those of the homologous genes found in other nematodes, which ranged from 924 to 979 bp for rrnL and 684 to 703 bp for rrnS [72] (Table S3). The A + T percentage of the *T. vitulorum* rrnS gene was 68.02%, and the rrnL gene had a higher A + T content (72.22%).

### 3.4. Variability and Informativeness of PCGs

**Figure 5:**
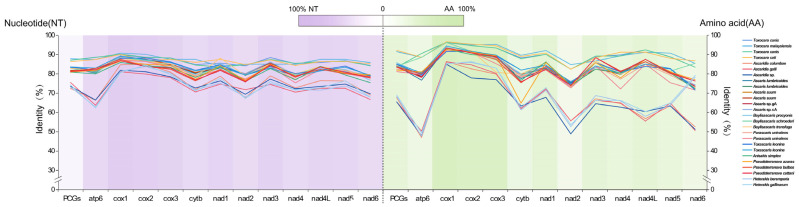
Pair-wise comparison of the nucleotide (**left**) and amino acid (**right**) sequence identities for PCGs. Colors from purple (high identity) to white (low identity) indicate the different identities of nucleotide sequences, and colors from green (high identity) to white (low identity) indicate the different identities of amino acid sequences.

The majority of the PCGs (atp6, cytb, cox3, nad1, nad3-5, and nad4L) of *T. vitulorum* mitogenome were identical to those of other *Toxocara* species in length (Table S3). Nevertheless, in order to further understand the evolution and divergence of *T. vitulorum* mitogenome, the sequence differences of the PCGs among Ascaridoidea species were analyzed in detail (Figure 5). The nucleotide sequence identities for each of the 12 PCGs of *T. vitulorum* ranged 60.4–91%. In addition, the amino acid sequence identities ranged 45.5–97.9%. Sequence identities of PCGs among the genus *Toxocara* were higher than others, followed by the genus *Toxascaris* and *Ascaris*, in contrast, the lowest sequence identities of PCGs were observed among the genus *Heterakis*. The highest nucleotide sequence and amino acid sequence identities were both observed in cox1 with a darker background color (Figure 5), while the lowest nucleotide sequence identities were observed and the lowest amino acid sequence identities were observed in nad2. The data indicated that cox1 (nucleotide sequence identity as 87.2% on average, amino acid sequence identity as 93.4% on average) was the most conserved gene, and the least conserved mitochondrial genes were nad6 (nucleotide sequence identity as 76.6% on average) and nad2 (amino acid sequence identity as 71.45% on average).

**Figure 6:**
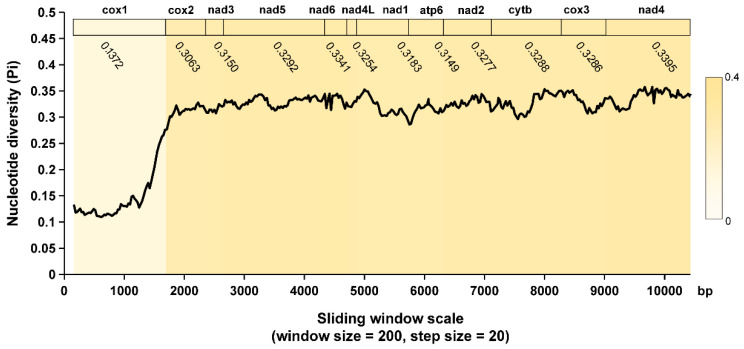
Sliding window analysis of the concatenated alignments of PCGs. The black line represents the value of the nucleotide diversity and are calculated using the parameters: window size = 200 bp and step size = 20 bp. Gene names, boundaries, and average nucleotide diversity values are indicated above the graph. Colors from yellow (high diversity) to white (low diversity) indicate the different nucleotide diversities.

Sliding window analysis was also employed in the PCGs of *T. vitulorum* and other Ascaridida species (Figure 6), to analyze the diversities within and between mitogenomes. It exhibited a highly variable nucleotide diversity among the concatenated 12 PCGs sequences of the mitogenomes, with Pi values for the 200 bp windows ranging from 0.137 to 0.334, and the Pi value of the whole concatenated alignment was 0.2988. The genes (cox2, cox3, cytb, atp6, nad1, nad2, nad3, nad4, nad4L, nad5, and nad6) all had relatively high nucleotide diversity of 0.306, 0.329, 0.329, 0.315, 0.318, 0.328, 0.315, 0.339, 0.325, 0.329, and 0.334, respectively, while gene cox1 had comparatively low nucleotide diversity of 0.137. Interestingly, across genes with high sequence variability, the genes with pronounced peaks and troughs of Pi seemed to have higher sequence variability than others, particularly nad4 and nad6. It appeared that cox1 was the least variable PCG, on the contrary, nad6 was one of the most variable PCGs, the conclusions were strikingly in line with pairwise comparisons between the PCG nucleotide and amino acid sequences from *T. vitulorum* mitogenome and those from the other reported Ascaridida nematodes. However, it appeared that nad4 also one of most variable PCG while it was nad2 observed in the identity analysis.

Cox1 was the least variable and slowly evolving mitogenome gene, according to pairwise comparisons and sliding window analysis of mitogenomes among Ascaridida mitogenomes. Further, the other PCGs exhibited significant nucleotide diversity and were rapidly developing genes, making them potential better molecular markers for species and population-level genetics investigations of *T. vitulorum*, particularly for diagnostics or detection involving cross contamination or other *Toxocara* species, since they could provide higher resolution for phylogenetically related taxonomic populations.

### 3.5. Substitution Ratios

**Figure 7:**
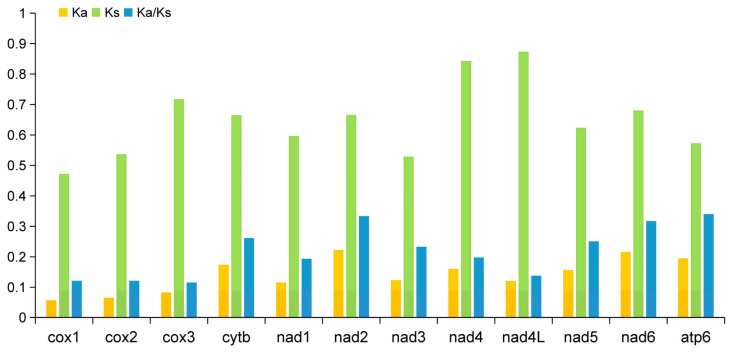
Evolutionary rates of PCGs between *T. vitulorum* and 28 other Rhabditida mitogenomes. The rates of non-synonymous substitutions (Ka) and synonymous substitutions (Ks) and the ratio of Ka/Ks are calculated for each PCG.

The Ka/Ks ratio could be used to measure the selective pressure that has been placed on PCGs during evolution. For the purpose of comprehending the dynamics of molecular sequence evolution, estimation of Ka and Ks substitution rates for the 12 PCGs was necessary [76]. The ratio is a good predictor that when Ka/Ks = 1, it indicates neutral mutation, when Ka/Ks > 1, it indicates positive or diversifying selection, and when Ka/Ks < 1, it indicates negative or purifying selection [77,78]. Ka, Ks, and the Ka/Ks ratio were calculated for 12 PCGs from *T. vitulorum* and 28 other nematode isolates (Figure 7). Of all 12 PCGs, the Ka/Ks ratios were <1, indicating that these genes underwent purifying or negative selection during their evolution. The Ka/Ks ratio analysis showed that cox1 (0.121), cox2 (0.121), and cox3 (0.116) were evolving under a strong purifying selection, whereas atp6 (0.340), nad2 (0.334), and nad6 (0.317) were evolving under comparatively relaxed mutational constraints.

### 3.6. Phylogenetic Analysis

**Figure 8:**
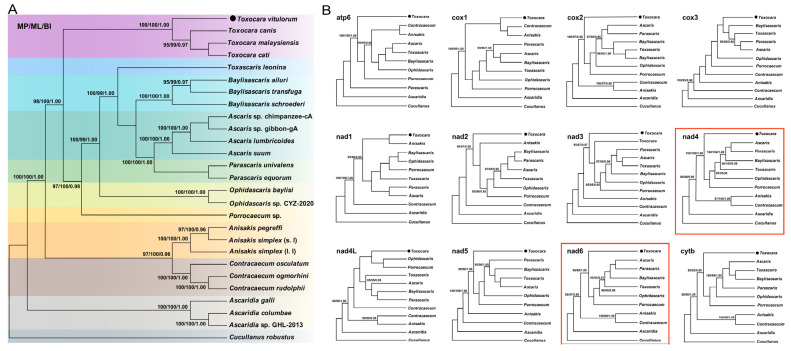
Phylogenetic relationships among Ascaridoidea nematodes. The phylogeny is inferred using the MP, ML, and BI methods of 27 Ascaridida species for which complete mitogenome sequences are available, using the Seuratoidea species (*C. robustus*) as the outgroup. (**A**) Phylogenetic tree is based on concatenated amino acid sequences of 12 PCGs. (**B**) Twelve topological phenograms are based on each PCG, and the tree topology consistent with the trees in A are marked with red line boxes. The black circle sign represents the species sequenced in this study. The numbers along the branches in A and B indicate bootstrap values/posterior probabilities (<95% or 0.95 supports not shown) resulting from different analyses in the order MP/ML/BI.

All three phylogenetic analyses (MP/ML/BI) using the concatenated amino acid data (Figure 8A) supported the phylogenetic relationships of *Ascaris*, *Ascaridia*, *Baylisascaris*, *Parascaris, Porrocaecum*, and *Toxascaris* (family Ascarididae); *Toxocara* (family Toxocaridae); *Anisakis*, *Contracaecum*, and *Ophidascaris* (family Anisakidae); *Ascaridia* (family Ascaridiidae); and *Cucullanus* (family Cucullanidae) in the order Ascaridida, and each genus was treated as a monophyletic sister group with high statistical supports (all statistical values ≥95 or 0.95). It was noteworthy that in Figure 8A, the three identical trees showed that *T. malaysiensis*, *T. cati,* and *T. canis* were more closely related to each other than to *T. vitulorum*, which supported a sister-species relationship of *T. vitulorum* with these three species in the family Toxocaridae. This finding was consistent with previous phylogenetic analyses based on the nuclear ITS-1 and ITS-2 and mitochondrial cox1 genes [23,24,79], but in contrast with the findings of Wickramasinghe et al. [30] and Li et al. [80], in which *T. vitulorum* was determined to more closely related to *T. malaysiensis/T. cati* than to *T. canis* and *Toxocara* was determined to more close related to *Porrocaecum* than to *Ascaris*/*Baylisascaris*/*Parascaris/Toxascaris* on the basis of several concatenated genes (e.g., 18S, 28S, cox2, and rrnS). It’s speculated that this might be attributed to use of incomplete or limited loci information. Perhaps, studies using mitogenome data from more large-scale species or isolates of *Toxocara* as well as taxa within the families Toxocaridae and Ascarididae worldwide are required to ascertain the phylogenetic relationships of *T. vitulorum* in the whole *Toxocara* and of the genera *Porrocaecum* and *Toxocara* in the superfamily Ascaridoidea of the order Ascaridida. Furthermore, the species of the family Toxocaridae were determined to be more closely related to species of the family Ascarididae than to species of the families Anisakidae, Ascaridiidae, and Cucullanidae, which was consistent with results of previous morphological and molecular studies [33,42,45,46,48,50,51,54,67,70,75,81,82,83,84] and also demonstrated the phylogenetic stability of the paraphyletic relationships characterized in the present study.

Subsequently, phylogenetic analyses were carried out using single protein-coding gene with an aim to screen out optimal genetic markers for phylogeny and molecular diagnostics in the order Ascaridida. As shown in Figure 8B, it appeared that although most genes showed different topologies, the phylogenetic positions of the genera *Ascaridia*, *Toxocara*, and *Cucullanus* were always stable in cox1-, cox2-, cox3-, cytb-, nad1-, nad2-, nad3-, nad4-, nad5-, and nad6-based phylogenetic analyses, as reported in other studies [45,55,56,85]. Further, compared to other 11 PCGs, the nad4 and nad6 genes shared the same topology as the genome-level phylogenetic analysis, suggesting that nad4 and nad6 among PCGs might be the best genetic markers and therefore could be instead of the mitogenome for molecular diagnostic, systematic, and evolutionary biological studies of this parasite and other related species in Ascaridida. Of course, the marker effectiveness of the nad4 and nad6 genes remains further validated when more additional Ascaridida mitogenomes become available, especially from the genus *Toxocara*, although concatenated mtDNA datasets are still the best molecular marker choice for evolutionary and phylogenetic studies of Ascaridida nematodes [33,42,45,46,48,50,51,54,67,70,75,81,82].

## 4. Conclusions

In this study, the complete mitogenome sequence of *T. vitulorum* was firstly determined and subjected to analyses and comparisons with other related species. Comparative genomics suggested that among the PCGs cox1 was the most conserved gene, whilst nad6 was the most varied gene. Genome- and individual gene-based phylogenies supported a sister-species relationship of *T. vitulorum* with the congeneric *T. canis*, *T. cati*, and *T. malaysiensis*. In further phylogenetic comparisons, the nad4 and nad6 genes were identified as novel genetic markers for phylogenetic and evolutionary studies of Ascaridida species. The results should contribute to the study of molecular diagnosis, prevention, and control of this parasitic nematode in future.

## Figures and Tables

**Table 1:** Summary of the mitogenome information included in this study.

Species	Host	Country	GenBank No.	References
*Anisakis pegreffii*	*Scomber japonicus*	Japan	LC222461	[42]
*Anisakis simplex* (s.l)	*Conger myriaster*	Korea	NC_007934	[43]
*Anisakis simplex* (s.s)	*Clupea harengusfrom*	Polanad	KC965056	[44]
*Ascaridia columbae*	Pigeon	China	JX624729	[45]
*Ascaridia galli*	Chicken	China	JX624728	[45]
*Ascaridia* sp. GHL-2013	Parrot	China	JX624730	[45]
*Ascaris lumbricoides*	Human	China	HQ704900	[46]
*Ascaris lumbricoides*	Human	Korea	NC_016198	[47]
*Ascaris* sp. (cA.)	*Hylobates hoolock*	China	KC839987	[48]
*Ascaris* sp. (gA.)	*Pan troglodytes*	China	KC839986	[48]
*Ascaris suum*	Pig	China	HQ704901	[46]
*Ascaris suum*	Pig	USA	NC_001327	[49]
*Baylisascaris ailuri*	*Ailurus fulgens*	China	HQ671080	[50]
*Baylisascaris procyonis*	*Procyon lotor*	China	JF951366	[51]
*Baylisascaris schroederi*	*Ailuropoda melanleuca*	China	NC_015927	[50]
*Baylisascaris transfuga*	*Ursus maritimus*	China	NC_015924	[50]
*Contracaecum ogmorhini*	Seal	Australia	KU558725	Unpublished
*Contracaecum osculatum*	*Clupea harengus*	Australia	NC_024037	[44]
*Contracaecum rudolphii*	Cormorant	China	NC_014870	Unpublished
*Cucullanus robustus*	*Conger myriaster*	USA	NC_016128	[52]
*Heterakis beramporia*	Chicken	China	KU529972	[53]
*Heterakis gallinarum*	Chicken	China	KU529973	[53]
*Ophidascaris baylisi*	*Python bivittatus*	China	MW880927	Unpublished
*Ophidascaris* sp.	*Elaphe carinata*	China	MK106624	Unpublished
*Parascaris equorum*	Horse	China	NC_036427	Unpublished
*Parascaris univalens*	Horse	China	NC_024884	[54]
*Pseudoterranova azarasi*	*Eumetopias jubata*	Japan	KR052144	[55]
*Pseudoterranova cattani*	*Otaria byronia*	Chile	KU558721	Unpublished
*Pseudoterranova krabbei*	*Halichoerus grypus*	Norway	KU558724	Unpublished
*Pseudoterranova bulbosa*	*Erignathus barbatus*	Canada	KU558720	Unpublished
*Porrocaecum* sp.	Crane	China	CNP0003131	[42]
*Toxascaris leonine*	Dog	Australia	KC902750	[56]
*Toxascaris leonine*	Cheetah	China	MK516267	Unpublished
*Toxocara canis*	Fox	Australia	EU730761	[57]
*Toxocara canis*	Dog	China	NC_010690	[33]
*Toxocara cati*	Cat	China	NC_010773	[33]
*Toxocara malaysiensis*	Cat	China	NC_010527	[33]

**Table 2:** The structure and organization of the mitogenome of *T. vitulorum*.

Gene	Location	Length	Start Codon	Stop Codon	Anticodon	Intergenic Region
trnN	1–59	59			GUU	0
trnY	60–120	61			GUA	−1
nad1	120–992	873	TTG	TAA		1
atp6	994–1591	598	ATT	T		0
trnK	1592–1652	61			UUU	0
trnL2	1653–1707	55			UAA	0
trnS1	1708–1760	53			UCU	0
nad2	1761–2605	845	ATT	TA		−1
trnI	2605–2666	62			GAU	0
trnR	2667–2721	55			ACG	1
trnQ	2723–2777	55			UUG	−1
trnF	2777–2837	61			GAA	0
cytb	2838–3944	1107	TTG	TAA		6
trnL1	3951–4005	55			UAG	0
cox3	4006–4773	768	TTG	TAG		0
trnT	4774–4845	72			UGU	0
nad4	4846–6075	1230	GTG	TAG		109
cox1	6185–7765	1581	TTG	TAG		2
trnC	7768–7822	55			GCA	8
trnM	7831–7890	60			CAU	1
trnD	7892–7947	56			GUC	−1
trnG	7947–8002	56			UCC	0
cox2	8003–8719	717	GTT	TAG		−10
trnH	8710–8765	56			GUG	0
rrnL	8766–9719	954				0
nad3	9720–10055	336	TTG	TAA		1
nad5	10057–11638	1582	ATG	T		0
trnA	11639–11693	55			UGC	2
trnP	11696–11750	55			UGG	0
trnV	11751–11806	56			UAC	0
nad6	11807–12241	435	TTG	TAA		−1
nad4l	12241–12472	232	ATT	T		0
trnW	12473–12533	61			UCA	−1
trnE	12533–12591	59			UUC	0
rrnS	12592–13279	688				13
trnS2	13293–13344	52			UGA	1701

## Data Availability

Molecular data have been deposited to GenBank with the following accession number: OP466744.

## Supplementary Materials

The following supporting information can be downloaded at: https://www.mdpi.com/article/10.3390/ani12243546/s1, Figure S1: Morphological photomicrographs of *Toxocara vitulorum* adults. (A) Total view of *T. vitulorum* adults, (B) Cephalic extremity with three lips and dentiform ridges, and (C) Caudal extremity; Figure S2: The proportion of the mitogenomes comprised by PCGs, tRNAs, rRNAs, and AT-rich and intergenic regions for *Toxocara* species; Table S1: List of the eight primer pairs for PCR amplification and their positions in the mitogenome of *Toxocara vitulorum*; Table S2: Nucleotide compositions of different genomic regions in *Toxocara vitulorum* and other nematodes reported within Ascaridida; Table S3: Length of each PCG in *Toxocara vitulorum* and other nematodes reported within Ascaridida.
