# Supplementary material for: The Complete Mitogenome of Toxocara vitulorum: Novel In-Sights into the Phylogenetics in Toxocaridae
Source: Animals (Basel). 2022 Dec 15;12(24):3546. doi: 10.3390/ani12243546 (PMC9774135; doi:10.3390/ani12243546)
Supplement: Supplementary file 1 [file animals-12-03546-s001.zip › Figures S1-S2.pdf]

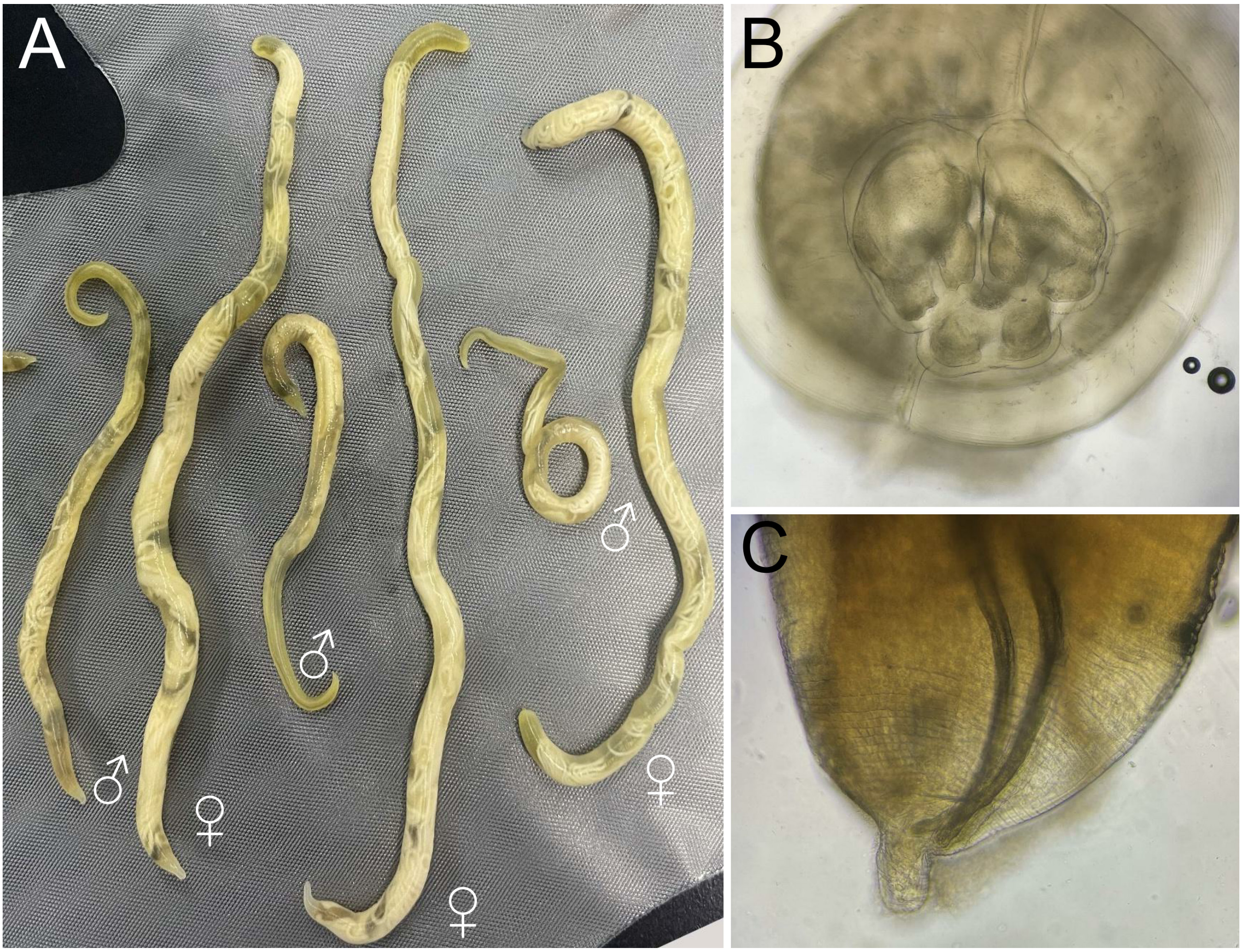

Figure S1. Morphological photomicrographs of *Toxocara vitulorum* adults. (A) Total view of *T. vitulorum* adults, (B) Cephalic extremity with three lips and dentiform ridges (10×), and (C) Caudal extremity (10×).

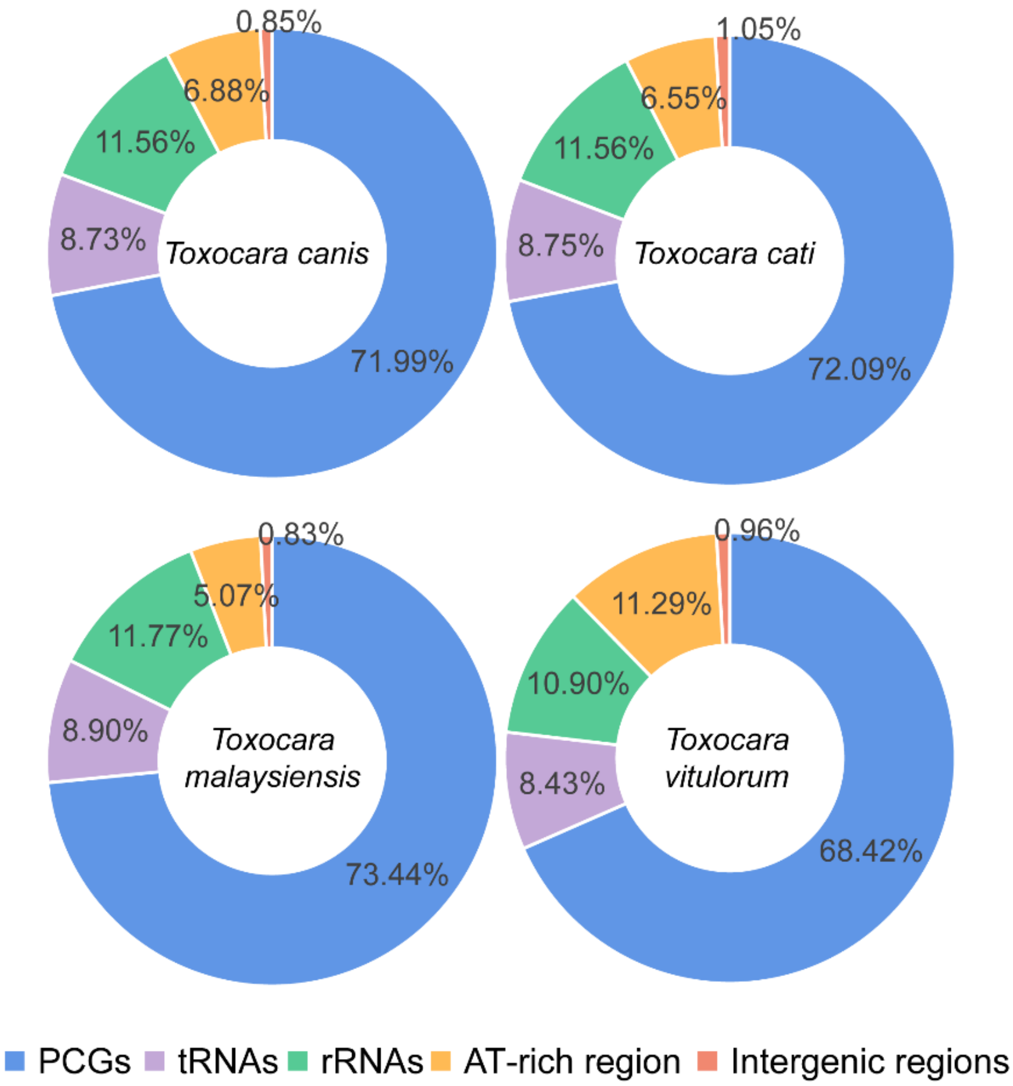

Figure S2. The proportion of the mitogenomes comprised by PCGs, tRNAs, rRNAs, and AT-rich and intergenic regions for *Toxocara* species.
