# Supplementary material for: The Complete Mitogenome of Toxocara vitulorum: Novel In-Sights into the Phylogenetics in Toxocaridae
Source: Animals (Basel). 2022 Dec 15;12(24):3546. doi: 10.3390/ani12243546 (PMC9774135; doi:10.3390/ani12243546)
Supplement: Supplementary file 1 [file animals-12-03546-s001.zip › Table S1.pdf]

**Table S1.** List of the eight primer pairs for PCR amplification and their positions in the mitogenome of *Toxocara vitulorum*

| **Primer name** | **Location based on *T. vitulorum* mitogenome** | **Primer sequence (5' to 3')** | |
|---|---|---|---|
| Tv-1 | 14907~14932 | Forward: | CACGAATGTGTACTAAATAATAAATA |
| | 1393~1404 | Reverse: | CGGTCAAAGCCAAAGG |
| Tv-2 | 1042~1069 | Forward: | CAGTTTTTGTTTTATTTGAAGGAAGGTA |
| | 2870~2897 | Reverse: | CTTTCTAGAAGGCAAACTAACAACCATA |
| Tv-3 | 2574~2598 | Forward: | GTTGTTCCCTTGACGGTGGTTTTTG |
| | 4772~4799 | Reverse: | CTAAAGCAGCCCAAACACAATACAACCT |
| Tv-4 | 4371~4390 | Forward: | TTTTGGGGTGCCGTTACTTA |
| | 7229~7253 | Reverse: | TAAACAGGAAAATAAAACCAAGCAC |
| Tv-5 | 6838~6868 | Forward: | GACTGATCGTAATTTGAATACTTCTTTTTTT |
| | 9560~9589 | Reverse: | TTACTAAAAGAAGAACAGTCTAAAACCCCA |
| Tv-6 | 9220~9240 | Forward: | GAGGACATTAAGGTAGCAAAA |
| | 11359~11379 | Reverse: | TAAAAAAGACAAAGGAGCAAA |
| Tv-7 | 10973~10996 | Forward: | AGCAGGATGGTCGTAATTATAGTA |
| | 13453~13475 | Reverse: | TTTCACAAATTTTTTTCGAGACA |
| Tv-8 | 13222~13242 | Forward: | GCCTATAGGGGAGTAAGTTGT |
| | 14778~15021 | Reverse: | ATAAGTAAAGGTATATAAGGGGTT |
