# Supplementary material for: The Complete Mitogenome of Toxocara vitulorum: Novel In-Sights into the Phylogenetics in Toxocaridae
Source: Animals (Basel). 2022 Dec 15;12(24):3546. doi: 10.3390/ani12243546 (PMC9774135; doi:10.3390/ani12243546)
Supplement: Supplementary file 1 [file animals-12-03546-s001.zip › Table S2.pdf]

Table S2. Nucleotide compositions of different genomic regions in *Toxocara vitulorum* and other nematodes reported within Ascaridida

Species		ALL	PCGs	tRNA	rRNA	3rd	cytb	cox1	cox2	cox3	nad1	nad2	nad3	nad4	nad4L	nad5	nad6	atp6
Ascaridia sp. GHL-2013	A+T%	67.100	66.150	66.880	67.580	67.000	65.310	65.790	64.050	66.180	65.400	69.420	66.670	67.150	70.040	65.590	69.650	66.670
	A-T skew	-0.398	-0.463	-0.170	-0.262	-0.433	-0.444	-0.373	-0.391	-0.517	-0.487	-0.511	-0.504	-0.438	-0.487	-0.528	-0.558	-0.492
	G+C%	32.900	33.850	33.120	32.420	32.900	34.690	34.210	35.960	33.830	34.600	30.580	33.330	32.850	29.950	34.410	30.350	33.340
	G-C skew	0.562	0.576	0.541	0.516	0.587	0.539	0.446	0.555	0.535	0.601	0.711	0.841	0.505	0.816	0.679	0.727	0.578
Anisakis simplex(s.l)	A+T%	71.200	69.540	71.900	76.070	71.800	67.880	66.120	66.950	66.320	68.160	73.290	75.300	69.910	76.730	72.060	71.260	70.670
	A-T skew	-0.357	-0.429	-0.174	-0.242	-0.421	-0.442	-0.413	-0.372	-0.492	-0.462	-0.442	-0.407	-0.421	-0.371	-0.414	-0.516	-0.401
	G+C%	28.800	30.460	28.090	23.930	28.400	32.120	33.880	33.050	33.680	31.840	26.720	24.700	30.090	23.280	27.940	28.730	29.330
	G-C skew	0.319	0.310	0.316	0.380	0.359	0.303	0.270	0.359	0.318	0.317	0.354	0.518	0.227	0.333	0.308	0.296	0.386
Anisakis simplex(s.s)	A+T%	70.745	69.660	69.750	76.100	71.198	66.670	66.110	67.090	66.890	67.930	73.870	75.590	69.350	78.450	73.010	72.640	70.500
	A-T skew	-0.362	-0.430	-0.182	-0.237	-0.415	-0.455	-0.409	-0.369	-0.487	-0.452	-0.456	-0.409	-0.421	-0.374	-0.411	-0.519	-0.409
	G+C%	29.255	30.340	30.250	23.910	28.802	33.340	33.890	56.650	33.110	32.080	26.120	24.400	30.650	21.560	27.000	27.360	29.500
	G-C skew	0.326	0.316	0.342	0.389	0.372	0.292	0.266	0.621	0.336	0.302	0.394	0.537	0.220	0.400	0.321	0.361	0.412
Ascaridia columbae	A+T%	71.100	70.240	71.170	71.830	71.900	68.670	68.770	67.390	69.940	67.920	72.360	72.030	71.920	76.960	72.610	73.100	70.350
	A-T skew	-0.280	-0.331	-0.079	-0.172	-0.252	-0.336	-0.269	-0.305	-0.405	-0.382	-0.377	-0.264	-0.303	-0.317	-0.328	-0.415	-0.390
	G+C%	28.900	29.750	28.830	28.170	28.000	31.330	31.220	32.610	30.060	32.080	27.640	27.970	28.070	23.040	27.390	26.900	29.650
	G-C skew	0.453	0.456	0.474	0.465	0.436	0.426	0.328	0.471	0.464	0.552	0.528	0.745	0.360	0.680	0.520	0.572	0.469
Ascaridia galli	A+T%	71.500	70.330	73.390	72.840	72.800	69.020	67.440	68.830	70.600	68.260	74.260	70.830	72.420	76.960	72.170	74.250	40.040
	A-T skew	-0.415	-0.479	-0.192	-0.279	-0.484	-0.470	-0.397	-0.403	-0.506	-0.539	-0.530	-0.588	-0.468	-0.437	-0.502	-0.585	-0.113
	G+C%	28.400	29.680	26.600	27.160	27.100	30.970	32.570	31.180	29.400	31.730	25.750	29.170	27.590	23.040	27.830	25.750	29.970
	G-C skew	0.486	0.496	0.489	0.467	0.587	0.450	0.360	0.484	0.525	0.525	0.640	0.816	0.443	0.760	0.572	0.661	0.463
Ascaris lumbricoides	A+T%	71.684	70.110	70.550	76.570	72.813	66.890	66.980	68.530	69.110	69.070	72.390	72.610	69.110	76.070	72.740	74.250	70.670
	A-T skew	-0.384	-0.474	-0.192	-0.284	-0.443	-0.504	-0.459	-0.378	-0.497	-0.489	-0.532	-0.500	-0.497	-0.438	-0.455	-0.511	-0.443
	G+C%	28.316	29.890	29.450	23.440	27.187	33.100	33.010	31.470	30.890	30.920	27.600	27.380	30.890	23.930	27.250	25.750	29.330
	G-C skew	0.440	0.450	0.401	0.467	0.554	0.455	0.394	0.464	0.400	0.422	0.562	0.761	0.400	0.750	0.472	0.446	0.500
Ascaris lumbricoides	A+T%	71.823	70.020	71.120	74.410	72.899	68.850	66.860	68.960	68.880	69.640	72.870	72.610	70.000	76.490	72.680	73.790	71.170
	A-T skew	-0.385	-0.473	-0.179	-0.214	-0.450	-0.497	-0.456	-0.369	-0.501	-0.490	-0.532	-0.500	-0.484	-0.430	-0.457	-0.520	-0.447
	G+C%	28.177	29.990	28.880	25.590	27.101	31.150	33.150	31.040	31.120	30.360	27.130	27.380	30.000	23.500	27.320	26.210	28.830
	G-C skew	0.446	0.456	0.424	0.407	0.493	0.479	0.392	0.456	0.406	0.441	0.589	0.761	0.355	0.746	0.474	0.456	0.526
Ascaris sp.(cA.)	A+T%	71.700	69.980	70.910	74.290	74.700	68.490	67.160	68.810	68.850	69.420	72.750	72.610	70.240	76.920	72.430	73.790	71.500

	A-T skew	-0.386	-0.473	-0.177	-0.214	-0.473	-0.503	-0.454	-0.376	-0.495	-0.492	-0.531	-0.500	-0.484	-0.422	-0.458	-0.520	-0.445
	G+C%	28.200	30.010	29.090	25.710	25.700	31.520	32.850	31.190	31.140	30.580	27.250	27.380	29.750	23.080	27.570	26.210	28.500
	G-C skew	0.447	0.455	0.423	0.405	0.525	0.480	0.390	0.468	0.389	0.438	0.582	0.761	0.361	0.741	0.469	0.456	0.532
Ascaris sp.(gA.)	A+T%	71.900	70.060	70.880	74.280	72.500	68.580	66.970	68.950	69.240	69.420	72.980	72.610	70.330	76.070	72.620	73.790	71.330
	A-T skew	-0.385	-0.473	-0.178	-0.213	-0.352	-0.501	-0.453	-0.373	-0.498	-0.495	-0.532	-0.500	-0.484	-0.438	-0.456	-0.520	-0.444
	G+C%	28.200	29.940	29.120	25.660	27.600	31.420	33.040	31.050	30.760	30.590	27.010	27.380	29.670	23.930	27.390	26.210	28.660
	G-C skew	0.447	0.456	0.418	0.413	0.384	0.478	0.382	0.465	0.406	0.446	0.596	0.761	0.364	0.750	0.470	0.456	0.523
Ascaris suum	A+T%	71.812	70.450	70.790	76.250	73.270	68.850	66.730	68.810	70.010	73.790	73.460	72.610	70.330	76.070	72.740	73.790	71.000
	A-T skew	-0.385	-0.473	-0.188	-0.284	-0.436	-0.503	-0.455	-0.372	-0.500	-0.520	-0.526	-0.500	-0.484	-0.438	-0.455	-0.520	-0.441
	G+C%	28.188	29.540	29.200	23.750	26.730	31.140	33.270	31.190	29.980	26.210	26.540	27.380	29.670	23.930	27.250	26.210	29.000
	G-C skew	0.452	0.465	0.418	0.465	0.539	0.491	0.387	0.459	0.434	0.456	0.607	0.761	0.364	0.750	0.472	0.456	0.506
Ascaris suum	A+T%	72.000	70.360	71.960	76.740	71.500	68.400	66.710	68.820	70.440	69.530	72.990	73.810	70.980	76.500	72.940	72.640	71.340
	A-T skew	-0.383	-0.470	-0.183	-0.280	-0.371	-0.505	-0.449	-0.380	-0.490	-0.483	-0.529	-0.484	-0.478	-0.441	-0.448	-0.513	-0.449
	G+C%	28.100	29.490	28.040	23.260	28.300	31.610	33.230	31.180	29.550	30.470	27.010	26.190	29.030	23.500	27.070	27.360	28.670
	G-C skew	0.452	0.460	0.434	0.462	0.456	0.481	0.378	0.477	0.427	0.436	0.596	0.772	0.367	0.782	0.459	0.395	0.535
Baylisascaris procyonis	A+T%	70.455	68.410	69.580	71.990	70.895	67.930	65.520	65.670	67.450	66.210	70.970	43.460	69.020	76.490	70.350	77.010	67.500
	A-T skew	-0.377	-0.483	-0.167	-0.229	-0.447	-0.508	-0.453	-0.420	-0.502	-0.498	-0.543	-0.178	-0.470	-0.419	-0.469	-0.504	-0.491
	G+C%	29.545	31.580	30.420	28.000	29.105	32.070	34.470	34.330	32.560	33.790	29.030	56.540	30.980	23.500	29.650	22.990	32.500
	G-C skew	0.449	0.466	0.383	0.434	0.524	0.476	0.397	0.483	0.392	0.464	0.608	0.842	0.354	0.746	0.498	0.480	0.549
Baylisascaris schroederi	A+T%	68.622	66.730	68.330	71.240	68.595	64.410	64.190	63.660	66.020	66.100	70.030	72.620	67.560	70.940	68.710	73.330	67.340
	A-T skew	-0.384	-0.486	-0.172	-0.318	-0.351	-0.506	-0.447	-0.416	-0.523	-0.484	-0.546	-0.484	-0.497	-0.482	-0.495	-0.549	-0.490
	G+C%	31.378	33.270	31.660	28.760	31.405	35.600	35.810	36.340	33.990	33.910	29.980	27.380	32.440	29.060	31.290	26.670	32.670
	G-C skew	0.457	0.475	0.445	0.509	0.325	0.462	0.402	0.488	0.425	0.466	0.620	0.717	0.348	0.706	0.500	0.431	0.592
Baylisascaris transfuga	A+T%	69.446	67.220	67.520	70.690	68.043	65.490	65.080	64.520	66.930	65.640	70.620	71.720	68.300	73.940	69.400	72.870	66.500
	A-T skew	-0.375	-0.478	-0.174	-0.230	-0.446	-0.490	-0.443	-0.415	-0.494	-0.473	-0.527	-0.452	-0.493	-0.457	-0.489	-0.539	-0.489
	G+C%	30.554	32.780	32.480	29.310	31.957	34.500	34.920	35.480	33.080	34.370	29.390	28.270	31.710	26.070	30.600	27.130	33.500
	G-C skew	0.447	0.463	0.401	0.432	0.444	0.456	0.397	0.508	0.409	0.447	0.605	0.642	0.348	0.705	0.505	0.390	0.552
Heterakis beramporia	A+T%	71.246	70.630	71.730	75.180	72.377	70.570	68.650	69.830	69.720	68.040	72.840	70.960	72.110	75.640	72.040	73.610	67.670
	A-T skew	-0.318	-0.376	-0.122	-0.259	-0.308	-0.362	-0.284	-0.317	-0.416	-0.413	-0.427	-0.409	-0.391	-0.424	-0.396	-0.434	-0.360
	G+C%	28.754	29.370	28.270	24.820	27.623	29.430	31.350	30.170	30.290	31.960	27.160	29.040	27.890	24.360	27.960	26.390	32.330
	G-C skew	0.462	0.474	0.449	0.561	0.485	0.392	0.314	0.419	0.500	0.500	0.624	0.815	0.423	0.684	0.570	0.632	0.381
Heterakis gallinarum	A+T%	69.842	69.310	70.760	74.110	72.407	68.880	67.820	67.250	68.320	65.750	72.600	70.960	70.490	72.220	70.270	74.080	67.840
	A-T skew	-0.336	-0.395	-0.151	-0.284	-0.338	-0.377	-0.272	-0.355	-0.412	-0.462	-0.444	-0.460	-0.400	-0.456	-0.429	-0.469	-0.388
	G+C%	30.158	30.690	29.240	25.890	27.593	31.120	32.190	32.760	31.690	34.240	27.410	29.040	29.520	27.780	29.720	25.920	32.160

	G-C skew	0.449	0.462	0.475	0.539	0.437	0.430	0.284	0.482	0.448	0.513	0.541	0.753	0.377	0.662	0.562	0.590	0.427
Parascaris equorum	A+T%	70.250	70.770	69.950	73.920	69.400	69.020	73.970	66.520	69.400	69.970	74.170	69.640	69.350	76.070	70.280	72.180	70.340
	A-T skew	0.384	-0.393	-0.152	-0.295	-0.366	-0.446	-0.518	-0.368	-0.490	-0.449	-0.483	-0.513	-0.451	-0.416	-0.436	-0.503	-0.498
	G+C%	29.800	29.230	30.040	26.070	30.200	30.980	26.030	33.480	30.590	30.020	25.830	30.360	30.650	23.930	29.710	27.820	29.670
	G-C skew	0.450	0.455	0.413	0.530	0.490	0.409	0.411	0.504	0.413	0.445	0.550	0.725	0.305	0.750	0.499	0.388	0.517
Parascaris univalens	A+T%	70.575	69.270	69.840	72.340	71.336	69.230	65.580	66.380	69.270	69.860	74.350	69.640	71.060	76.070	70.670	72.640	70.340
	A+T%	-0.385	-0.449	-0.156	-0.222	-0.443	-0.449	-0.423	-0.366	-0.477	-0.451	-0.481	-0.513	-0.471	-0.416	-0.445	-0.506	-0.498
	A-T skew	29.425	30.730	30.160	27.660	28.664	30.770	34.420	33.620	30.720	30.130	25.640	30.360	28.940	23.930	29.330	27.360	29.670
	G+C%	0.454	0.471	0.432	0.448	0.481	0.407	0.415	0.498	0.398	0.447	0.551	0.725	0.309	0.750	0.508	0.428	0.517
Pseudoterranova azarasi	A+T%	70.711	69.420	71.530	75.240	69.963	67.520	64.970	67.090	68.800	68.270	73.880	72.920	70.250	76.730	70.830	74.710	69.500
	A-T skew	-0.369	-0.439	-0.151	-0.258	-0.352	-0.433	-0.422	-0.359	-0.457	-0.483	-0.443	-0.478	-0.437	-0.393	-0.440	-0.514	-0.424
	G+C%	29.289	30.580	28.470	24.760	30.037	32.480	35.030	32.900	31.200	31.730	26.120	27.080	29.760	23.270	29.170	25.290	30.500
	G-C skew	0.342	0.347	0.345	0.435	0.363	0.291	0.297	0.351	0.339	0.307	0.412	0.671	0.284	0.519	0.368	0.491	0.366
Pseudoterranova cattani	A+T%	71.075	69.850	71.830	75.130	69.800	66.600	65.550	68.520	69.580	68.610	74.350	75.300	70.000	75.860	71.840	73.790	70.670
	A-T skew	-0.362	-0.431	-0.155	-0.251	-0.289	-0.433	-0.423	-0.351	-0.441	-0.439	-0.437	-0.431	-0.435	-0.409	-0.439	-0.489	-0.434
	G+C%	28.925	30.150	28.470	24.870	30.100	33.390	34.460	31.490	30.410	31.390	25.650	24.700	30.000	24.140	28.160	26.210	29.330
	G-C skew	0.336	0.350	0.333	0.418	0.289	0.348	0.319	0.348	0.313	0.299	0.392	0.687	0.263	0.572	0.354	0.403	0.432
Pseudoterranova krabbei	A+T%	70.343	68.960	71.160	70.230	69.171	41.380	64.720	66.950	67.630	68.270	73.520	75.000	69.410	74.570	70.450	73.560	69.830
	A-T skew	-0.352	-0.445	-0.147	-0.124	-0.324	-0.114	-0.441	-0.357	-0.463	-0.470	-0.460	-0.445	-0.436	-0.410	-0.448	-0.500	-0.446
	G+C%	29.657	31.050	28.830	29.770	30.829	33.940	35.280	33.050	32.380	31.730	26.480	25.000	30.410	25.440	29.540	26.440	30.160
	G-C skew	0.343	0.355	0.313	0.244	0.350	0.358	0.342	0.345	0.306	0.300	0.446	0.690	0.267	0.491	0.342	0.426	0.414
Pseudoterranova bulbosa	A+T%	71.240	69.930	71.270	75.630	69.776	66.150	65.920	67.810	68.800	68.500	74.110	74.410	70.400	76.290	72.230	75.860	71.170
	A-T skew	-0.363	-0.435	-0.152	-0.239	-0.283	-0.435	-0.446	-0.353	-0.438	-0.451	-0.439	-0.448	-0.427	-0.401	-0.439	-0.491	-0.438
	G+C%	28.760	30.070	28.730	24.370	30.224	33.850	34.070	32.190	31.200	31.500	25.890	25.600	29.590	23.700	27.780	24.140	28.830
	G-C skew	0.345	0.357	0.335	0.425	0.310	0.341	0.352	0.381	0.297	0.309	0.379	0.674	0.269	0.527	0.359	0.409	0.457
Toxascaris leonine	A+T%	71.537	70.220	71.150	74.790	72.453	69.140	67.430	68.240	69.400	68.500	73.220	73.810	70.000	74.360	71.680	74.480	71.160
	A-T skew	-0.387	-0.475	-0.165	-0.312	-0.387	-0.490	-0.449	-0.409	-0.508	-0.468	-0.485	-0.492	-0.475	-0.437	-0.480	-0.549	-0.471
	G+C%	28.463	29.780	28.850	25.210	27.547	30.870	32.580	31.760	30.600	31.500	26.780	26.190	30.000	25.640	28.320	25.520	28.840
	G-C skew	0.458	0.475	0.420	0.480	0.443	0.473	0.393	0.469	0.447	0.433	0.628	0.772	0.382	0.700	0.491	0.477	0.572
Toxascaris leonine	A+T%	71.100	70.100	70.990	74.250	71.800	68.950	66.600	67.670	68.360	67.470	71.560	71.720	70.080	76.920	72.120	75.400	70.330
	A-T skew	-0.378	-0.469	-0.164	-0.326	-0.421	-0.473	-0.446	-0.412	-0.493	-0.498	-0.503	-0.494	-0.462	-0.389	-0.479	-0.531	-0.474
	G+C%	29.000	29.880	29.010	25.760	28.300	31.050	33.400	32.330	31.640	32.540	28.440	28.280	29.920	23.080	27.880	24.600	29.670
	G-C skew	0.428	0.460	0.411	0.474	0.442	0.412	0.381	0.469	0.416	0.465	0.600	0.705	0.348	0.666	0.456	0.495	0.528
Toxocara canis	A+T%	68.424	67.260	69.340	71.860	68.810	66.220	65.240	65.130	65.240	64.850	70.150	72.730	67.730	71.120	69.480	73.560	68.900

	A-T skew	-0.367	-0.455	-0.156	-0.289	-0.381	-0.462	-0.489	-0.368	-0.489	-0.511	-0.500	-0.458	-0.431	-0.418	-0.438	-0.494	-0.495
	G+C%	31.576	58.390	30.580	27.490	31.190	33.780	34.770	34.870	34.770	35.140	29.620	27.270	32.280	28.880	30.520	26.440	31.110
	G-C skew	0.404	0.676	0.401	0.457	0.517	0.449	0.393	0.438	0.393	0.381	0.472	0.778	0.350	0.522	0.426	0.339	0.484
Toxocara canis	A+T%	68.600	67.690	68.510	72.030	69.000	65.760	63.240	64.990	65.370	65.410	70.060	71.720	67.970	68.730	66.860	72.810	70.820
	A-T skew	-0.362	-0.395	-0.153	-0.293	-0.420	-0.467	-0.429	-0.371	-0.490	-0.503	-0.497	-0.452	-0.433	-0.494	-0.103	-0.500	-0.406
	G+C%	31.400	32.310	31.490	27.970	30.800	34.240	36.760	35.010	34.640	34.590	29.940	28.270	32.030	31.270	33.140	27.190	29.190
	G-C skew	0.401	0.414	0.411	0.448	0.487	0.446	0.379	0.432	0.398	0.384	0.462	0.726	0.345	0.476	0.351	0.322	0.500
Toxocara cati	A+T%	69.948	70.820	70.190	71.530	70.188	68.200	64.510	67.510	67.320	67.010	70.980	73.510	68.210	72.850	71.430	74.940	70.230
	A-T skew	-0.363	-0.406	-0.135	-0.219	-0.466	-0.430	-0.415	-0.312	-0.466	-0.477	-0.506	-0.449	-0.445	-0.432	-0.434	-0.479	-0.476
	G+C%	30.052	29.190	29.800	28.470	29.812	31.800	35.480	32.480	32.690	32.990	29.030	26.490	31.790	27.160	28.570	25.060	29.770
	G-C skew	0.392	0.500	0.387	0.404	0.349	0.397	0.339	0.403	0.355	0.396	0.510	0.730	0.304	0.524	0.416	0.413	0.461
Toxocara malaysiensis	A+T%	68.900	68.150	70.290	70.050	68.900	65.130	64.390	65.120	66.020	66.670	71.680	71.730	67.310	72.850	70.100	74.940	69.400
	A-T skew	-0.370	-0.396	-0.116	-0.345	-0.422	-0.473	-0.422	-0.382	-0.475	-0.471	-0.488	-0.411	-0.437	-0.467	-0.455	-0.491	-0.494
	G+C%	31.200	31.850	29.720	29.950	30.600	34.870	35.610	34.880	33.990	33.330	28.320	28.270	32.680	27.150	29.890	25.060	30.600
	G-C skew	0.410	0.420	0.392	0.440	0.444	0.435	0.354	0.444	0.410	0.389	0.456	0.726	0.338	0.587	0.455	0.413	0.530
Toxocara vitulorum	A+T%	69.942	66.930	68.980	72.220	68.116	65.940	65.460	66.240	67.450	64.720	68.280	70.830	66.590	72.850	72.100	71.030	67.060
	A-T skew	-0.370	-0.488	-0.164	-0.315	-0.375	-0.515	-0.440	-0.394	-0.506	-0.526	-0.529	-0.488	-0.482	-0.456	-0.533	-0.540	-0.526
	G+C%	30.058	33.070	30.940	27.780	31.884	34.050	34.540	33.750	32.550	35.280	31.710	29.160	33.420	27.150	27.900	28.970	32.940
	G-C skew	0.445	0.458	0.410	0.426	0.470	0.501	0.359	0.487	0.480	0.422	0.478	0.776	0.406	0.619	0.533	0.397	0.523
