# Supplementary material for: The Complete Mitogenome of Toxocara vitulorum: Novel In-Sights into the Phylogenetics in Toxocaridae
Source: Animals (Basel). 2022 Dec 15;12(24):3546. doi: 10.3390/ani12243546 (PMC9774135; doi:10.3390/ani12243546)
Supplement: Supplementary file 1 [file animals-12-03546-s001.zip › Table S3.pdf]

Table S3. Lengths of each PCG and rRNA in *Toxocara vitulorum* and other nematodes reported within Ascaridida

**Species**	**atp6**	**cox1**	**cox2**	**cox3**	**cytb**	**nad1**	**nad2**	**nad3**	**nad4**	**nad4L**	**nad5**	**nad6**	**rrnS**
Ascaridia sp. GHL-2013	597	1561	687	751	1101	870	837	339	1230	217	1575	435	691
Anisakis simplex(s.l)	600	1576	699	755	1099	873	846	336	1230	232	1582	435	699
Anisakis simplex(s.s)	600	1576	699	764	1101	876	846	336	1230	232	1582	435	700
Ascaridia columbae	597	1563	696	745	1101	876	843	336	1236	217	1581	435	703
Ascaridia galli	597	1563	699	745	1104	876	843	336	1236	217	1581	435	701
Ascaris lumbricoides	600	1578	699	777	1098	873	844	336	1230	234	1585	435	703
Ascaris lumbricoides	600	1578	699	768	1098	873	844	336	1230	234	1585	435	700
Ascaris sp.(cA.)	600	1577	699	777	1098	873	844	336	1230	234	1585	435	700
Ascaris sp.(gA.)	600	1577	699	777	1098	873	844	336	1230	234	1585	435	700
Ascaris suum	600	1578	699	777	1098	873	844	336	1230	234	1585	435	699
Ascaris suum	600	1577	699	768	1098	873	844	336	1230	234	1585	435	701
Baylisascaris procyonis	600	1578	699	768	1107	873	844	336	1230	234	1585	435	700
Baylisascaris schroederi	600	1578	699	768	1107	873	844	336	1230	234	1585	435	694
Baylisascaris transfuga	600	1578	699	768	1107	873	844	336	1230	234	1585	435	694
Heterakis beramporia	600	1563	696	766	1128	876	843	334	1230	234	1581	432	699
Heterakis gallinarum	597	1563	696	767	1128	876	843	334	1230	234	1581	432	699
Parascaris equorum	600	1578	699	768	1104	876	844	336	1230	234	1585	435	700
Parascaris univalens	600	1569	699	768	1095	876	850	336	1230	234	1582	435	702
Pseudoterranova azarasi	600	1576	702	766	1102	873	846	336	1230	232	1584	435	702
Pseudoterranova cattani	600	1576	702	766	1102	873	846	336	1230	232	1584	435	702
Pseudoterranova krabbei	600	1576	702	766	1102	873	846	336	1230	232	1584	435	702
Pseudoterranova bulbosa	600	1576	702	766	1102	873	846	336	1230	232	1584	435	702
Toxascaris leonine	600	1578	699	768	1095	873	844	336	1230	234	1582	435	700
Toxascaris leonine	600	1578	699	768	1095	873	844	336	1230	234	1582	435	700
Toxocara canis	598	1578	714	768	1107	882	844	330	1230	232	1576	435	693
Toxocara canis	598	1578	714	768	1107	873	845	336	1230	233	1582	434	697
Toxocara cati	598	1578	711	768	1107	873	844	336	1230	232	1582	435	696
Toxocara malaysiensis	598	1581	711	768	1107	873	844	336	1230	232	1582	435	696
Toxocara vitulorum	598	1581	717	768	1107	873	845	336	1230	232	1582	435	688
